# Malaria – a major health problem within an oil palm plantation around Popondetta, Papua New Guinea

**DOI:** 10.1186/1475-2875-8-56

**Published:** 2009-04-08

**Authors:** Bianca Pluess, Ivo Mueller, Damien Levi, Graham King, Thomas A Smith, Christian Lengeler

**Affiliations:** 1 Swiss Tropical Institute, PO Box, 4002 Basel, Switzerland; 2 Institute of Medical Research, PO Box 60, Goroka, Papua New Guinea; 3 CTP (PNG) Ltd., Higaturu Oil Palms, PO Box 28, Popondetta, Papua New Guinea

## Abstract

**Background:**

For companies operating in malaria endemic countries, malaria represents a substantial risk to workers and their dependants, and can lead to significantly reduced worker productivity. This study provides an overview of the malaria epidemiology within an oil palm plantation in Popondetta, south-eastern Papua New Guinea, its implication for the company with its employees and their families and the potential for control.

**Methods:**

In 2006, we carried out a cross-sectional study within six company villages, which included the determination of parasite rates by conventional microscopy, interviews and haemoglobin measurements. Passive surveillance data were collected from the 13 company aid posts for the years 2005 and 2006.

**Results:**

Malaria prevalence was found to be high: all-age prevalence was 33.5% (95% CI 30.1–37.0) in 723 individuals. *Plasmodium falciparum *was the dominant species, followed by *Plasmodium vivax *and *Plasmodium malariae*. Children between five and nine years of age were most affected (40.3%, 95% CI 0.32–0.49). Haemoglobin levels were found to be low; 11.0 g/dl (95% CI 10.8–11.1) for men and 10.4 g/dl (95% CI 10.3–10.5) for women, respectively. *Plasmodium falciparum *infections were significantly associated with anaemia (Hb < 10 g/dl). At the aid posts, all malaria cases in 2005 and January-March 2006 were diagnosed by symptoms only, while from April 2006 onwards most cases were tested by rapid diagnostic tests. Between 2005 and 2006, 22,023 malaria cases were diagnosed at the aid posts and malaria accounted for 30–40% of all clinical cases. Of the malaria cases, 13–20% were HOP employees. On average, an employee sick with malaria was absent for 1.8 days, resulting in a total of 9,313 workdays lost between 2005 and 2006. Sleeping outside of the house did not increase the risk of a malaria infection, neither did getting up before 7 am.

**Conclusion:**

Malaria was found to be a major health burden in the Higaturu Oil Palm plantation, posing a high risk for company staff and their relatives, including expatriates and other non-immune workers. Reducing the malaria risk is a highly recommended investment for the company.

## Background

Papua New Guinea (PNG) is characterized by its variability and complexity in culture, ecology and geography. This complexity is also reflected in the malaria situation [1]. Malaria ranks first amongst the diseases causing illness and death in Papua New Guinea [2], although there is a great variation in the relative importance of malaria for people in different areas. The epidemiology of malaria in PNG ranges from complete absence of malaria, through unstable low levels of transmission with recurring epidemics, to permanently high levels of transmission [3,4], even reaching the highest transmission levels known outside of Africa. About 46% of all Papua New Guineans live in an altitude zone of 0 – 600 m above sea level [1], where malaria is highly endemic.

Malaria represents not only a health, but also an economic burden. In Africa alone, the estimated direct and indirect costs of malaria exceed US$ 12 billion annually [5]. Malaria negatively influences economic growth, reducing it for example by 1.3% annually in endemic countries between 1965 and 1990. In 1995, the average gross domestic product in non-malarious countries was five times higher than in countries affected with malaria [6]. The most immediate effect of malaria on a company is its impact on the workforce and the resulting cost of caring for sick employees. Employees sick with malaria are not working efficiently and are likely to take time off to recover. In a worst case, employees die and replacements need to be recruited and trained [7].

In PNG, oil palm is a major agro-industry. In 2001, over 100,000 hectares of oil palms were planted. Since 2000, palm oil has been the most important agricultural export industry in PNG, with nearly 400,000 tons exported in 2002, amounting to approximately 136 million USD [8,9]. Commercial plantations such as oil palm plantations offer suitable environmental conditions for *Anopheles *mosquitoes breeding and people living and working in such plantations are thus likely to be at high risk for malaria [10]. However, such plantations also provide relatively good housing and infrastructure for their workers, making it relatively easy to deploy screening or indoor residual spraying, and improvement in case management.

Higaturu Oil Palms plantations (HOP) is one of the major employers in Oro Province (south-east PNG). It provides housing for its employees and their dependants within the plantation. HOP is located in the area around Popondetta, the capital of Oro province, on a coastal plain at the foot of the Owen-Stanley range. So far, and to the best of our knowledge, no data have ever been collected on the epidemiology of malaria for this part of the country. Therefore, this study aims to provide (1) an overview of the malaria epidemiology in Oro province, and (2) to quantify the problem of malaria within a commercial oil palm plantation, its implications for the company and the potential for control. Few such studies have been carried out and this economic risk (in addition to the health risk) urgently requires to be better quantified.

## Methods

### Study site

The study was conducted within the oil palm plantations of CTP (PNG) Ltd. (trading as Higaturu Oil Palms – HOP), close to Popondetta, the capital of Oro Province in Papua New Guinea. HOP processes palm fruits from an area of about 22,997 hectares. The company owns 8,997 hectares which are divided into five estates (namely Javuni, Sumbiripa, Ambogo, Mamba and Embi). Smallholders own another 14,000 hectares. While the smallholders are responsible for maintaining and harvesting the palms, Higaturu buys their fruits and processes them into palm oil and palm kernel oil.

People living in the 14 villages within the plantation consist of HOP employees and their dependants, for whom housing is provided by the company. Every village is equipped with a small health centre, staffed with one Community Health Worker or nursing officer. A bigger health centre with a laboratory is situated in Siroga village, which is easily accessible by car or foot.

The cross-sectional study took place in six villages (namely Epa, Irigi, Sumbiripa, Irihambo, Javuni and Moale), located within three of the five estates. Two of the estates (Mamba and Embi) were excluded as they were difficult to reach due to their geographical distance. Mamba estate is located in the mountains and hence is not ecologically comparable to the other estates. For the passive surveillance system, all aid posts were included.

**Figure 1:**
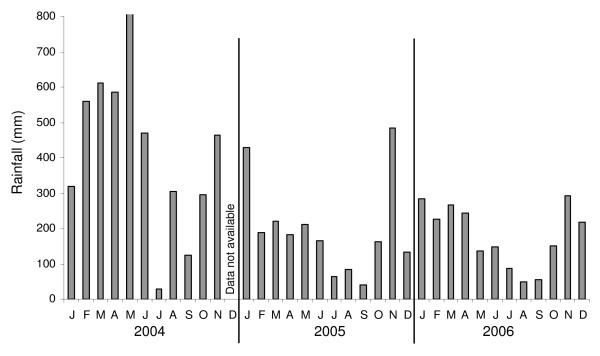
**Average monthly rainfall within the study area from 2004 to 2006**.

Ecologically the study villages are very similar to each other, all being surrounded by oil palms. Within the study area, rainfall usually shows some seasonality with a wet season from November to April and a drier season from May/June to October. During our study period the average rainfall was 189 mm, with 243 mm in April and 135 mm in May (Figure 1).

**Figure 2:**
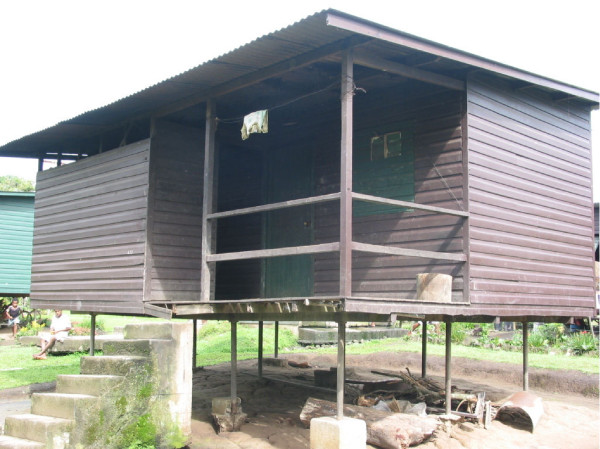
**A typical worker's house**.

A company village consists of 100 houses of which 87–98% are so-called "labour houses", wooden two-bedroom houses with a cooking place outside on the veranda and its own ablution block and toilet next to the house (Figure 2).

### Cross-sectional parasitology surveys

The cross-sectional surveys were conducted between April and May 2006. Every village was visited once. Households to be included were selected by random sampling from the existing list of houses provided by the company.

#### Inclusion/exclusion criteria

Every person living within the selected villages and households was eligible for the study. Consent procedures are described below. Persons ill at the time of the survey, as well as visitors to the household who had lived less than four weeks in the village, were excluded from the study.

#### Measurements

From each participant we measured the axillary temperature with an electronic thermometer. At the same time, a questionnaire was administered on the use of bed nets, the history of malaria sickness in the previous two weeks, the use of anti-malarial drugs and health facility attendance, travel behaviour, evening leisure activities and sleeping habits (outside or inside their house). The interview was performed in English or Melanesian pidgin by bilingual interviewers. At the same time as the interview, a blood slide including a thick and a thin film was prepared. Thin blood films were fixed with methanol. All smears were stained with 2.5% buffered Giemsa (pH 7.2) for 35 minutes and examined afterwards microscopically for 100 fields under oil immersion (× 1000 magnification) before being declared negative. In positive films, parasite species were identified and densities recorded as the number of parasites/200 white blood cells (WBC). Densities were converted to the number of parasites/μl of blood assuming 8,000 WBC/μl.

Haemoglobin levels were measured from the same finger prick as from the blood slide, using a HaemoCue machine (HemoCue B-Hemoglobin, Angholm, Sweden). From each individual the spleen size was determined according to Hackett's grading by a local nurse.

### Routine data assessment

Malaria incidence data routinely collected by the health staff of the HOP aid posts were available from January 2005 to December 2006. Until April 2006, all patients presenting with fever were considered as malaria cases. In April 2006 Rapid Diagnostic Tests (RDTs, ICT Malaria Combo Cassette, R&R Marketing, South Africa) were introduced in all aid posts within the Higaturu Oil Palm estates. Before the introduction of the RDTS, a three-day training course on the theory and usage of the RDTs was provided to every member of the health staff of HOP. After the introduction of RDTs, patients were classified as having malaria only if the RDT result was positive. Compared to microscopy the RDT sensitivity was 89%, which is excellent.

For every patient visiting a HOP aid post, the date of the visit, data of joining the company (employee, dependant or outsider) and the diagnosis were collected.

In 2006, for an ad hoc sample of employees, the numbers of days missing from work due to malaria were recorded.

### Data entry and analysis

The data from the questionnaires were entered in an Access 2002 database (Microsoft Corp., Redmond, US). To check for data entry mistakes, the entered data were checked and corrected by one person, while a second person was reading out the entries of the original questionnaires. The blood slides were read twice by two different microscopists and the results entered into two different Excel databases (Microsoft Corp., Redmond, US). A Kappa test for checking for consistency between the two datasets showed an expected agreement of 78.4% (k = 0.43). In case one microscopist only found few parasites (< 3 parasites/200WBC) and the other none, the slides were considered to be positive. Slide results were excluded from the analysis if one microscopist found a high parasites density (>12 parasites/200 WBC) and the second one found none. Differences in categorical variables were tested using the Pearson chi-square test. Differences were regarded as significant if the p-value of the test statistic was ≤5%.

Changes of haemoglobin values were calculated using linear regression, adjusting for age, gender and mosquito net use. Difference between males and females in haemoglobin levels were tested with Wilcoxon test, as the Bartlett's test for the differences in the variance was significant. Participants were classified as having anaemia if haemoglobin levels were under 10 g/dl. For obtaining odds ratios, relevant variables (age, sex, village, sleep outside on the veranda, sleep outside in a shelter, sleeping under a bed net, work and getting up before 7 am) were added one-by-one in the model and a likelihood ratio tests was performed to test for significance.

Work days lost for the company were calculated by taking the arithmetic mean of the days not worked per employee due to malaria.

Unfortunately, it was not possible to get the results of RDT testing from all the clinics because these statistics were not available to us. In order to have at least an estimate of testing positivity rate, a sample of 90 successive patients in one clinic (Siroga) who were clinically diagnosed with malaria were also tested with a RDT in order to calculate the RDT positivity rate.

All statistical analyses were performed in Intercooled Stata 9 (StataCorp, Texas, US).

### Informed consent

The community was informed about the aims and the methods of the study before the start of the data recording and sample collection by the company health care workers. Consent was then sought individually from all study participants or their guardians. This was done by explaining again the aim and procedure of the study at the beginning of the interview. A signature of each participant on their understanding and willingness to participate was then collected. All enrolled individuals retained their right to withdraw from the study at any stage.

The survey methodology was approved by the PNG Medical Research Advisory Committee (approval number MRAC No. 06.07).

## Results

### Cross-sectional survey: general description

A total of 843 individuals were included in the survey (Table 1). The study participants consisted of 423 (50.2%) females and 419 (49.8%) males and were grouped into the following five age categories: 0–4, 5–9, 10–19, 20–39 and >40 years. There was a big excess of individuals aged 20–39 years (40.9% of the participants), which were made up largely of workers, as expected in this population. Over half of the participants (57.1%) came from Oro province, and overall 81.7% of the people had an Oro, a Morobe or a mixed Oro/Morobe origin. 28.2% (238/843) of the interviewee were permanently employed by the company and of those, 89.4% (211/236) were working within the field department. Employees within the field department work in the plantations, mainly harvesting ripe palm fruits. Only 6.8% (57/843), of the participants were hired as casuals, of whom 87.7% (50/57) were women.

**Table 1:** Description of cross-sectional survey participants

**Variable**		**N (%)**	**Male**	**Female**
Sex	Male	423 (50.2)		

	Female	419 (49.8)		

	Total	842		

Age	0–4	169 (20.1)	89 (21.1)	79 (18.9)

	5–9	148(17.6)	76 (18.0)	72 (17.2)

	10–19	117 (13.9)	47 (11.1)	70 (16.7)

	20–39	344 (40.9)	167 (39.6)	177 (42.2)

	>40	64 (7.6)	43 (10.2)	21 (5.0)

	Total	842	422	419

Origin	Oro	480 (57.1)	218 (51.8)	261 (62.4)

	Morobe	160 (19.1)	97 (23.0)	63 (15.1)

	Oro/Morobe	46 (5.5)	24 (5.7)	22 (5.3)

	Other provinces	157(18.3)	82(19.5)	72(17.2)

	Total	840	421	418

Work	Permanent	238 (28.2)	205 (86.1)	33 (13.9)

	Casual	57 (6.8)	7 (12.3)	50 (87.7)

	Not working	548 (65.0)	211 (38.6)	336 (61.4)

	Total	843	423	419

Work department	Field	211 (89.4)	178 (87.7)	33 (100.0)

	Security	12 (5.1)	12 (5.9)	0 (0.0)

	Transport	7 (3.0)	7 (3.5)	0 (0.0)

	Mill	4 (1.7)	4 (2.0)	0 (0.0)

	Growers	1 (0.4)	1 (0.5)	0 (0.0)

	HR	1 (0.4)	1 (0.5)	0 (0.0)

	Finance	0 (0.0)	0 (0.0)	0 (0.0)

	Total	236	203	33

Work casual	Collect mama lusfrut	22 (38.6)	0	22 (44.0)

	Weeding	13 (22.8)	1 (14.3)	12 (24.0)

	Empty Fruit Bunch (EFB) distribution	8 (14.0)	1 (14.3)	7 (14.0)

	Fertilizer application	6 (10.5)	0 (0.0)	6 (12.0)

	Harvest helper	3 (5.3)	2 (28.6)	1 (2.0)

	Other	5 (8.8)	3 (42.9)	2 (4.0)

	Total	57	7	50

Population movement was low with only 7.9% (66/839) individuals reporting to have been travelling within the last four weeks. 70.9% of 839 participants slept under a mosquito net the previous night. However, many people reported, that the treatment status of their nets was poor, with many of the nets not having been retreated within one year of usage.

### Parasitological results

**Figure 3:**
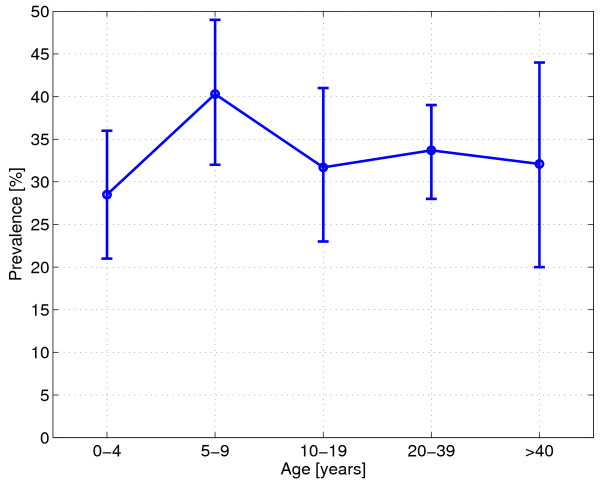
**Malaria prevalence (all species) by age group, with 95% confidence intervals**.

**Figure 4:**
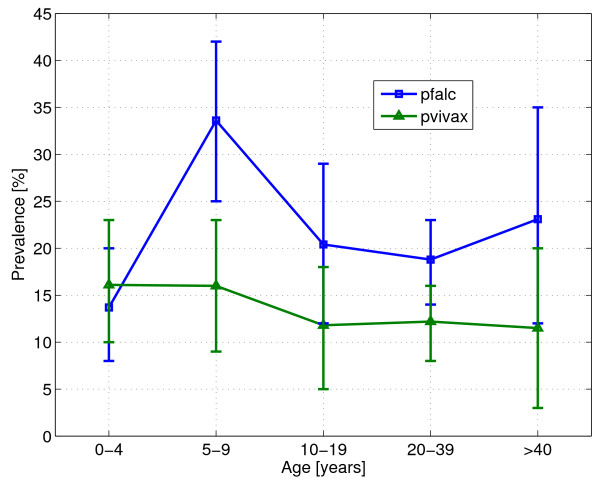
**Malaria prevalence by species and age group, with 95% confidence intervals**.

A total of 723 blood slides were collected, of which 242 were positive for malaria (33.5%, 95% confidence interval [CI]: 30.1–37.0). This result documents the high level of endemicity in this area. Between the three estates, the malaria prevalence differed significantly (χ^2 ^= 24.85, 2 df, p < 0.001), ranging from 20.3% (95% [CI] 0.14–0.26) in Ambogo to 43.1% (0.37–0.49) in Sumbiripa (Table 2). At village level, the malaria prevalence ranged from 17.7% (95% CI 0.09–0.27) in Epa to 44.6% (95% CI 0.36–0.53) in Sumbiripa village (χ^2 ^= 25.80, 5 df, p < 0.001) (Table 2). There was no significant difference between prevalence among males and females (χ^2 ^= 0.10, 1 df, p = 0.319). Overall, prevalence was highest in the age group 5–9 years (40.3%, 95% CI 0.32–0.49) (Figure 3). The same pattern was seen with *P. falciparum *infections, with 27.9% (95% CI 0.20–0.36) of the 5 to 9 years old children infected (Figure 4). For *P. vivax*, no difference between the age-groups was found (Figure 4).

**Table 2:** Malaria prevalence and bed net usage, by estates and by villages (cross-sectional survey).

**Estate**	**Number examined**	**Prevalence all species n infected; % (95% CI)**	**Prevalence *P. falciparum *n infected; % (95 % CI)**	**Prevalence *P. vivax *n infected; % (95% CI)**	**Bed net usage (%)**
**Ambogo**	177	36; 20.3 (0.14–0.26)	28; 15.8 (0.10–0.21)	6; 3.4 (0.01–0.06)	84.3

**Sangara**	279	91; 32.6 (0.27–0.38)	56; 20.0 (0.15–0.25)	35; 12.5 (0.09–0.16)	69.7

**Sumbiripa**	267	115; 43.1 (0.37–0.49)	60; 22.5 (0.17–0.27)	50; 18.7 (0.14–0.23)	63.3

**Village**					

**Epa**	68	12; 17.7 (0.09–0.27)	9; 13.2 (0.05–0.21)	2; 2.9 (-0.01–0.07)	97.5

**Irigi**	109	24; 22.0 (0.14–0.30)	19; 17.4 (0.10–0.25)	4; 3.7 (0.00–0.07)	75.8

**Moale**	154	48; 31.2 (0.24–0.38)	28; 18.1 (0.12–0.24)	23; 14.8 (0.09–0.20)	65.6

**Javuni**	125	43; 34.4 (0.26–0.43)	28; 22.4 (0.15–0.30)	12; 9.6 (0.04–0.15)	75.0

**Irihambo**	137	57; 41.6 (0.33–0.50)	33; 24.1 (0.17–0.31)	21; 15.3 (0.09–0.21)	57.6

**Sumbiripa**	130	58; 44.6 (0.36–0.53)	27; 20.8 (0.14–0.28)	29; 22.3 (0.15–0.29)	68.9

With 59.5% (144/242) of all positives, *P. falciparum *was the dominant species, followed by *P. vivax *(91/242, 37.6%) and *P. malariae *(16/242, 6.6%). No *Plasmodium ovale *was found. A high number of the positive slides (26/242, 10.7%) were mixed infections. The parasite densities of the infections were low, with 91.1% (194/213) of the slides having <500 parasites/μl. No difference in parasite densities between age groups was found (χ^2 ^= 20.18, 12 df, p = 0.064). When investigating differences by species, 11.1% (14/144) of *P. falciparum *infections were >500 parasites/μl, compared to 4.4% (4/91) of the *P. vivax *infections (significant difference χ^2 ^= 10.48, 2 df, p = 0.005).

### Reported malaria episodes and spleen rates

25 participants presented with fever at the survey and a quarter (26.1%) of the 842 participants reported having had malaria during the past two weeks. No correlation between measured or reported fever and malaria parasitaemia was found: eight of the 20 (40.0%) people presenting with fever and having a blood slide taken, had a positive blood slide (χ^2 ^= 0.41, 1 df, p = 0.521). Of the participants with reported fever during the last two weeks and having a malaria slide taken, only 37.6% (70/186) had a positive slide (χ^2 ^= 1.86, 1 df, p = 0.173).

Almost everybody who reported having had malaria within the last two weeks visited a health centre (99.5%) and took anti-malarials (96.2%).

Overall, 59.7% of the participants had an enlarged spleen with an average size of 3.2 on the Hackett scale, but no association with a current malaria infection was seen, neither at individual (χ^2 ^= 0.78, 1 df, p = 0.377) nor at village level. Looking at age groups, the average Hackett score was lowest for the youngest age group (0–4 years) with 2.4, and it rose with age to 3.8 for the oldest age group (>40 years). Despite the fact that this was reported differently by Genton *et al*. within the Wosera [3], a high rate of enlarged spleens in adults is a particular feature of malaria in PNG. Often, enlarged spleens in adults are linked to hyperreactive malarial splenomegaly [1]. Though usually present within mid altitude zones [1], this syndrome might also occur within this study area.

Haemoglobin levels were low with a significantly higher level for men (11.0 g/dl, 95% CI 10.8–11.1) than for women (10.4 g/dl, 95% CI 10.3–10.5) (χ^2 ^= 24.73, 1 df, p < 0.001). In children aged 5–9 years, the haemoglobin levels were significantly decreased with a malaria infection: by 1.0 g/dl (95% CI -1.43 – -0.47, p < 0.001) for any species and 1.2 g/dl (95% CI -1.66 – -0.63, p < 0.001) for *P. falciparum *infections. A significant association between *P. falciparum *and anaemia was found (χ^2 ^= 4.41, 1 df, p = 0.036).

### Behavioural aspects

There was no difference between employees and dependants in the risk of getting infected with malaria (Table 3). Neither were sleeping outside during the night (on the veranda or on a shelter) nor getting up before 7 am associated with a higher risk of a malaria infection. On an individual level, sleeping under a bed net showed no protection against malaria (OR 1.2, 95% CI 0.8–1.7). However, there was a clear trend towards less malaria with higher bed net coverage between villages (Table 2). For every percentage increase in net coverage, the odds of malaria cases decreased by 3% (OR 0.97, 95% CI 0.96–0.99).

**Table 3:** Risk factors for getting a malaria infection (cross-sectional survey).

**Variable**	**Total (n)**	**Infected (%)**	**Odds ratio**	**CI 95%**	**p-value (LRT)**
Village					

Moale	154	48 (31.2)	1		0.00

Epa	68	12 (17.7)	0.5	0.2–1.0	0.00

Irigi	109	24 (22.0)	0.6	0.3–1.1	

Javuni	125	43 (34.4)	1.1	0.7–1.9	

Irihambo	137	57 (41.6)	1.5	0.8–2.6	

Sumbiripa	130	58 (44.6)	1.2	1.1–3.2	

Sex			0.9	0.6–1.3	0.51

Age					

0–4	130	37 (28.5)	1		0.14

5–9	129	52 (40.3)	1.9	1.1–3.2	

10–19	101	32 (31.7)	1.1	0.6–2.0	

20–39	306	103 (33.7)	1.2	0.6–2.3	

>40	56	18 (32.1)	1.0	0.4–2.4	

Work permanent (vs not working (= dependants))	215	74 (34.4)	1.0	0.5–1.8	1.00

Work casual (vs not working (= dependants))	50	18 (36.0)	1.0	0.5–2.1	

Sleep under a bed net (yes vs no)	507	160 (31.6)	1.2	0.8–1.7	0.39

Sleep on veranda (yes vs no)	136	44 (32.4)	1.1	0.4–1.3	0.56

Sleep in a shelter (yes vs no)	63	25 (39.7)	0.7	0.4–1.3	0.25

Get up before 7 am (vs getting up after 7 am)	538	181 (33.6)	0.9	0.8–1.1	0.51

### Incidence of routinely diagnosed malaria infections

**Figure 5:**
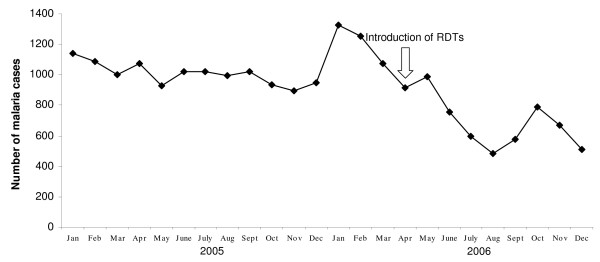
**Monthly routine malaria incidence data from 2005 to 2006**.

Malaria was the most common disease diagnosed by HOP health staff in 2005 and 2006. In 2005 all malaria patients seen at the aid posts were diagnosed by clinical symptoms only. Of all patients seen at the 13 HOP aid posts in 2005, 39% (12,083/30,864) were diagnosed with malaria. Of the 12,083 malaria patients, 26% (3159/12083) were employees. On average, 1007 patients were diagnosed with malaria every month at the 13 aid posts. Malaria cases were found to be evenly distributed throughout the year 2005 (Figure 5), hence showing no seasonality.

In 2006, fewer patients were diagnosed with malaria than in 2005, especially after the introduction of RDTs in April. The RDT positivity rate, calculated for a sample of 90 patients, was found to be 48%. Overall, 9,940 patients were sick with malaria, still representing 29% (9,940/34,216) of all diagnosis made at the aid posts. 20% (2,015/9,940) of the malaria patients were employees. The malaria burden was highest in the beginning of the year, and then, after the introduction of RDTs in April a sharp decline until August was seen. During the three months in 2006 before the introduction of RDTs, 1,220 malaria cases were clinically diagnosed every month, of which 17% (211/1,220) were employees. After the introduction of RDTs in April and until the end of 2006, the monthly average of overall malaria cases decreased to 698. 153 (22%) of these malaria cases were employees. Malaria cases showed a seasonal peak in October, which did not reach the level of 2005, before RDT introduction (Figure 5).

### Days lost for the company

According to HOP administration, an employee sick with malaria was absent from work for an average of 1.8 days. Since 3,159 employees were sick with malaria in 2005 and 2,015 employees in 2006, this resulted in 5,686 lost days in 2005 and 3,627 lost days in 2006.

## Discussion

To our knowledge, this work presents one of the few publications on the impact of malaria within an agro-industrial operation in endemic countries and the first one in PNG. Furthermore, it provides for the first time malariological information for the region around Popondetta, south-east PNG.

Clearly, malaria was found to be a major health problem within the CTP Plantation. The overall prevalence rate of 33.5% found among workers and their families is high. Furthermore, children and adults are both affected and this suggests there is little acquired immunity. This level of endemicity is similar to that found in other surveys done in the lowlands of PNG outside agro-industrial operations. Genton *et al *[3] and Cattani *et al *[11] found prevalence rates of 60% in the Wosera (East Sepik) and 35.0% to 42.7% surrounding Madang, respectively. The prevalence data of Genton *et al *and Cattani *et al *have been collected between 10 and 20 years ago and it is likely that the overall rate of transmission has decreased since. Furthermore, an oil palm plantation represents an artificial environment with its own ecosystem, which is likely to have a different transmission rate than a natural habitat. Comparisons with other oil palm plantations within PNG on the same altitudes would have been interesting, but such data are not available. Given the particular malaria epidemiology of PNG, which is by far the highest in the region, comparison with other Asian and Pacific countries is meaningless.

HOP offers housing to their employees within the geographical boundaries of the plantation. This special setting, with only employees and their dependants being allowed to live within the plantation, resulted in 40% of the participants being aged between 20 and 39. This is clearly not representative for the general population of the country and hence caution needs to be exerted when generalizing our results to the rest of the population.

Ripe palm fruits are collected by company trucks, creating deep wheel tracks on the ground. With enough rainfall such tracks present perfect breeding sites for *Anopheles punctulatus *[12], which were found by human landing catches within the study area (Cooper R.D. personal communication). Though we conducted only one cross-sectional survey, the age peak for malaria infections (5–9 years old) was found to be characteristic for a highly endemic area [1,3]. Such an intense malaria transmission can pose a high risk for all residents, especially for expatriates and other non-immune workers (for example coming from the cooler highlands). In one of the few documented examples, a large joint venture in Mozambique lost 13 expatriate employees due to malaria within two years [13]. Undoubtedly, malaria endemicity weighs down the attractiveness of industrial or agro-industrial sites.

Almost everybody who was feeling sick with presumptive malaria within the last two weeks had visited a health centre and received anti-malarials. This finding was not surprising, as employees and their dependants have free and ready health services provided by the company. This good access to treatment explains that we have not come across of any reported deaths from malaria.

In 2005, with the exception of one clinic, all malaria cases were diagnosed purely on a symptomatic basis. Unfortunately, symptoms of malaria are non-specific and many other diseases can present with the same clinical picture, including harmful diseases such as dengue, HIV or hepatitis B. But also many harmless viral infections present similar to a malaria infection. Thus, a purely symptomatic diagnosis of malaria can lead to a vast over-diagnosis of malaria cases [14-16]. This was illustrated by the fact that after the introduction of RDTs in April 2006 the number of reported malaria cases decreased steadily. But even with systematic testing with RDTs, still about 10,000 patients were diagnosed with malaria in 2006. This high level of morbidity represents a considerable cost of treatment (estimated cost per treatment: USD 0.18) and a substantial loss of productivity. These episodes resulted in 5686 lost days for the company in 2005, amounting to lost wages of over USD 60,000 (King G., personal communication). In 2006, the company lost 3627 working days due to malaria. The costs associated with malaria illness will increase significantly as PNG moves towards introducing the significantly more expensive Coartem^® ^(artemether-lumefantrine) as the national first-line treatment.

More than half of the infections were caused by *P. falciparum *(57.4%), followed by *P. vivax *with 36.2%. *Plasmodium falciparum *infections are well known for their impact on morbidity and mortality, but also *P. vivax *infections are considered to be responsible for a substantial health burden [15,17-19]. For an oil palm plantation, where approximately 60–75% of the employees do a physically strenuous work, a healthy workforce is crucial for a high productivity. The negative impact of malaria is also demonstrated by the low average haemoglobin level, which is a further drain on the energy of workers.

Virtually all inhabitants within the study area live within so-called "labour houses" (Figure 2), wooden two-bedroom houses, in which room temperatures can get very high, including at night. As a result, people sometimes sleep outside on their veranda or in a self-built shelter, where they are fully exposed to biting mosquitoes. Surprisingly, sleeping on a veranda or sleeping on a shelter was not found to be a risk-factor for a malaria infection. The most likely explanation for this finding is that the mosquitoes prevalent within the plantation, *Anopheles punctulatus *(RD Cooper, personal communication) are exo- but also endophagic [20] and can easily enter and bite occupants inside the non mosquito-proofed houses.

The two main tools to prevent malaria infections are insecticide-treated mosquito nets (ITNs) and indoor residual spraying (IRS). Within HOP, nets are sold for half their commercial price, which explains the high usage rate of 70%. On an individual level no significant protection against malaria could be detected but this is expected in a situation with a homogeneously high level of net use. The village comparison showed a trend of decreasing number of malaria infections with increasing bed net level, confirming that the nets actually do have an impact. Unfortunately, the insecticide re-treatment level of the nets was poor since most nets were not retreated after 2004. Therefore, the introduction of LLINS, which are designed to maintain their efficacy against mosquitoes for at least three years without any re-treatment necessary, would be highly recommended [21]. The high level of net protection also suggests that endemicity would be even higher if there was no protection.

Indoor residual spraying requires 1) an adequate knowledge of vector behaviour and occurrence, and (2) a highly structured programme including well-trained personnel, properly used insecticides, good logistics and scheduling and a high level of sustained financing [22]. These terms would clearly be doable by a commercial oil palm plantation and thus, IRS would also be a feasible option for malaria control in the HOP setting. With housing provided by the plantation, upgrading the housing conditions including house screening would be a more long-term solution of malaria control. This approach has already shown great success within military barracks in Pakistan and India, reducing the malaria incidence up to 72% [23].

## Conclusion

Malaria was found to be a major health burden to the Higaturu Oil Palms, posing a high risk for all inhabitants, indigenous workers as well as non-immune workers. Detrimental effect included direct cost of treatment, days away from work and reduced physical ability. These losses are substantial enough to warrant the implementation of energetic and long-lasting malaria control measures.

## Competing interests

GK was the General Manager of the Higaturu Oil Palms plantations (HOP) from March 2005 to October 2007. DL is employed as Health Extension officer at HOP. BP, IM, TS and CL declare that they have no competing interests.

## Authors' contributions

BP participated in the design of the study, conducted the field work, analysed and interpreted the data and drafted the manuscript. IM and CL were involved in designing and implementing the study and writing of the manuscript. TS assisted in the data analysis and writing of the manuscript. DL and GK were facilitating the overall coordination of the fieldwork and helped with the acquisition of the data. All authors read and approved the final manuscript.
